# Mycotoxins in Raw Bovine Milk: UHPLC-QTrap-MS/MS Method as a Biosafety Control Tool

**DOI:** 10.3390/toxins15030173

**Published:** 2023-02-24

**Authors:** Marta Leite, Andreia Freitas, Jorge Barbosa, Fernando Ramos

**Affiliations:** 1 Faculty of Pharmacy, Health Science Campus, University of Coimbra, Azinhaga de Santa Comba, 3000-548 Coimbra, Portugal; 2 National Institute for Agricultural and Veterinary Research (INIAV), Rua dos Lágidos, Lugar da Madalena, 4485-655 Vila do Conde, Portugal; 3 Associated Laboratory for Green Chemistry (LAQV) of the Network of Chemistry and Technology (REQUIMTE), R. D. Manuel II, 4051-401 Porto, Portugal

**Keywords:** regulated mycotoxins, emerging mycotoxins, milk, UHPLC-MS/MS, QuEChERS, validation

## Abstract

Mycotoxins are compounds produced by several fungi that contaminate agricultural fields and, either directly or by carry-over, final food products. Animal exposure to these compounds through contaminated feed can lead to their excretion into milk, posing threats to public health. Currently, aflatoxin M1 is the sole mycotoxin with a maximum level set in milk by the European Union, as well as the most studied. Nonetheless, animal feed is known to be contaminated by several groups of mycotoxins with relevance from the food safety point of view that can be carried over into milk. To evaluate the multi-mycotoxin occurrence in this highly consumed food product it is crucial to develop precise and robust analytical methodologies towards their determination. In this sense, an analytical method for the simultaneous identification of 23 regulated, non-regulated, and emerging mycotoxins in raw bovine milk using ultra-high-performance liquid chromatography coupled with tandem mass spectrometry (UHPLC-MS/MS) was validated. A modified QuEChERS protocol for extraction purposes was used, and further validation was performed by assessing the selectivity and specificity, limits of detection and quantification (LOD and LOQ), linearity, repeatability, reproducibility, and recovery. The performance criteria were compliant with mycotoxin-specific and general European regulations for regulated, non-regulated, and emerging mycotoxins. The LOD and LOQ ranged between 0.001 and 9.88 ng mL^−1^ and 0.005 and 13.54 ng mL^−1^, respectively. Recovery values were between 67.5 and 119.8%. The repeatability and reproducibility parameters were below 15 and 25%, respectively. The validated methodology was successfully applied to determine regulated, non-regulated, and emerging mycotoxins in raw bulk milk from Portuguese dairy farms, proving the importance of widening the monitoring scope of mycotoxins in dairy products. Additionality, this method presents itself as a new strategic and integrated biosafety control tool for dairy farms for the analysis of these natural and relevant human risks.

## 1. Introduction

Milk constitutes an important source of micro- and macronutrients in the human diet, which provides to this highly worldwide consumed food product its beneficial health properties [1,2]. World milk production is mainly represented by cow milk (81%), and it is expected to grow in the next decade at a higher rate than most of the main agricultural commodities [3]. An increase in global consumption is equally expected, with vulnerable age groups as major consumers [3,4]. Nonetheless, raw milk consumption has also been growing, since it has been considered, especially among health-conscious people, as having higher health benefits, which can be reduced via industrial processing. These consumption patterns can also lead to the exposure of eminent hazards due to the presence of food contaminants, such as mycotoxins, which can be carried over, biotransformed, and secreted to milk during animal metabolism due to the consumption of naturally contaminated feed [5]. This mycotoxic contamination occurs from the basis and throughout the food chains due to the presence of mycotoxigenic fungi, mainly from the *Aspergillus*, *Penicillium*, *Fusarium*, and *Alternaria* genera [4]. This exposure can lead to severe human health effects comprising damage at the DNA and nerve level, immune deficiency, and cancer, with possible increased negative effects due to co-occurrence patterns [6,7,8].

Aflatoxin M1 (AFM1), a hydroxylated metabolite of aflatoxin B1 (AFB1) and a class 1 human carcinogen, is considered the most representative mycotoxic contaminant in milk [9]. Due to its high toxicity and thermal stability, the European Commission has set maximum levels for AFM1 in milk, namely for raw milk, heat-treated milk, and milk for the manufacture of milk-based products at 0.05 µg kg^−1^, and for infant formulae and follow-on formulae (infant milk and follow-on milk) at a 0.025 µg kg^−1^ [10]. Although most studies aim at the determination of AFM1 in this foodstuff, representative mycotoxins in animal feed systems should also be considered, since one of the main routes of milk contamination is through animal mycotoxic exposure via contaminated feed and feed ingredients [11,12]. Mycotoxins reported as of public health importance include aflatoxins (AFs), citrinin (CIT), deoxynivalenol (DON), fumonisins (FBs), HT-2 toxin, nivalenol (NIV), ochratoxin (OTA), T-2 toxin, and zearalenone (ZEA), which are regulated by the European Commission concerning feed and feed ingredients, namely maize silage, the most predominant component in feed systems [13,14]. The main toxic effects on human health caused by these groups of mycotoxins include, for example, liver cancer and hepatocellular carcinoma by AFs, immunotoxicity and gastroenteritis by DON, renal diseases by OTA, and esophageal cancer and neural tube defects by FBs [15]. Nonetheless, in recent years, emerging mycotoxins have become a hot topic [16]. This exponentially growing group of mycotoxins includes beauvericin (BEA), enniatins (ENNs) such as enniatin A (ENNA) and enniatin B (ENNB), moniliformin (MON), and tenuazonic acid (TEA), and are frequent toxic compounds found in feed and its individual components [1,17,18]. Additionally, the number of reports concerning multiple exposures to mycotoxins in milk has been increasing over the years, especially due to co-occurrence patterns that can lead to synergistic or additive effects [18,19,20].

To comply with official regulations and to respond to the changing mycotoxic occurrence patterns, the need for accurate, precise, sensitive, and robust analytical methodologies is of high importance for the quantitative analysis of mycotoxins at low levels [21,22]. Multi-analyte techniques have become an excellent tool to respond to the wide range of these chemical compounds in single matrices [23,24]. Commonly applied methods for the determination of mycotoxins are based on rapid methods, such as ELISA, lateral flow devices, biosensors, and fluorescence polarization immunoassay (FPIA), and chromatographic methods, including gas chromatography coupled to mass spectrometry (CG-MS), thin-layer chromatography (TLC), high-performance liquid chromatography coupled to ultraviolet (UV), diode-array or fluorescence detectors, and liquid chromatography coupled to mass spectrometry-based methods (LC-MS) [24,25,26]. Currently, ultra-high-performance liquid chromatography coupled with tandem mass spectrometry (UHPLC-MS/MS) has become the gold-standard method and the most extensively used for the determination of the different chemical families of mycotoxins due to its higher sensitivity, specificity, and efficient ability compared to other analytical techniques [21,27,28]. The multi-analysis of mycotoxins also represents a challenge because of the complexity of contaminated matrices and, consequently, the selection of proper preparation and extraction procedures [29,30]. Different strategies have been used for the clean-up and extraction of mycotoxins from various commodities, which include the dilute-and-shoot approach (DaS), solid-phase extraction (SPE), SIDA methods, immunoaffinity columns (IAC), and the QuEChERS (Quick Easy Cheap Effective Rugged Safe) approach, with the latter increasingly being used for this purpose due to its simplicity, minimal and greener clean-up, and fast extraction and purification [29,31,32]. This original pesticide clean-up protocol consists of applying acetonitrile (ACN) as an extraction solvent followed by the addition of magnesium sulfate (MgSO_4_) and sodium chloride (NaCl) as a partitioning step, and a mixture of salts and sorbent materials (primary–secondary amine (PSA), C18, C8, alumina, or others) as a dispersive solid-phase extraction (dSPE) for additional purification [29,33,34]. This final step contributes to reduced matrix effects, resulting in satisfactory recoveries of the analytes, which allow its application to mycotoxin determination in food- and feed-based samples with a relative rate of success with slight modifications depending on the chemical range of the compounds of interest, and on matrix composition for increased recovery yields [34,35]. Overall, QuEChERS methodology represents an enhancement in the throughput of mycotoxin monitoring, while being economically cost-effective. 

The main objective of the current work was to validate a precise and robust analytical methodology for the determination of regulated, non-regulated, and emerging mycotoxins in raw bovine milk, comprising a promising biosafety control tool for mycotoxigenic risk assessment procedures. The selected mycotoxins were based on a previous comprehensive evaluation performed to define the representative toxic compounds encountered in animal feed contamination with possible carry-over to milk [4]. The development of this method encompassed the evaluation of performance criteria, including selectivity and sensitivity, limits of detection and quantification (LOD and LOQ), linearity, repeatability, reproducibility, and recovery, according to the European guidelines for acceptability of method validation. Application to real samples as an outcome provided insights on the importance of monitoring of a wider range of mycotoxins in milk samples with significant potential in food safety and public health.

## 2. Results and Discussion

Multi-exposure to mycotoxins in contaminated animal feed can lead to the excretion of parent mycotoxins or their metabolites into milk, with severe consequences for human health. Approximately 10% of worldwide milk samples present AFM1 in levels above the maximum set by the EC [20]. Nonetheless, other studies have reported the presence of FBs (FB1 and B2), AFs (AFB1, B2, G1, and G2), OTA, and ZEA [36,37]. Although several groups of mycotoxins fit in this scenario, the current methods to determine mycotoxins in milk are mainly based on the determination of AFM1 alone, or on the aforementioned mycotoxins targeted as a low number of analytes per method (fewer than six).

In this study, a previously extensively optimized methodology for the detection of regulated and emerging mycotoxins (*n* = 23) in maize grains was applied and re-validated for milk matrices [38]. Standardization of the extraction and detection methods on multiple matrices is one of the goals of the authors, aiming at a full comprehensive study of the whole maize value chain and the identification of crucial contamination stages until the final product, namely milk. The QuEChERS protocol fulfilled the criteria for this purpose, being a current widely and extensively used clean-up/extraction protocol for multi-mycotoxin determination in several feed and food matrices due to its advantages compared to other clean-up/extraction procedures [39,40,41,42,43,44,45]. Specific applications of this protocol to milk samples is nonetheless scarce. For example, Michlig et al. [21] used QuEChERS for the determination of only one mycotoxin (AFM1) in milk samples, with representative performance criteria regarding maximum levels established by EU validation guidelines [46]. A similar approach for the determination of AFM1 and AFB1 was also used by Rodríguez-Carrasco et al. [47]. A recently published method for the determination of 40 mycotoxins in raw milk samples, including regulated and emerging compounds, includes a protocol of 10 mL of an initial sample with AME, BEA, CTA, CTN, FA, Hydro-FB1, OTA, RC, STC, FBs, and ENNs being analyzed with a QuEChERS protocol without a dispersive solid-phase extraction (dSPE) step; and analysis of the other mycotoxins using a complete protocol. 

The number of analytes analyzed in this study in a less-time consuming and greener approach gives this technique a relative rate of success by combining LLE with the salting-out and dSPE steps characteristic of the QuEChERS protocol [25,29,35,48]. The authors were also able to successfully develop this method in milk samples using low quantities of initial samples (4 mL) and low quantities of QuEChERS materials with comparable results to those of other published protocols [39]. The present method, characterized by a complete QuEChERS method based on a C18 sorbent for dSPE, was further combined with the gold-standard analytical method of UHPLC-MS/MS, which is a recognizable, highly sensitive, and precise quantitative method allied with the capacity of low detection levels and polarity switching for single runs of chemically different compounds [49]. Representative feed chain mycotoxins, namely MON, NIV, penicillic acid (PA), TEA, and tentoxin (TTX), were also for the first time analyzed in milk samples. 

For validation purposes, preliminary studies were first performed by analyzing raw milk samples collected in dairy farms and submitted to the previously optimized and validated analytical method [38] towards the search of blank matrices. The application of the validation process to samples not contaminated with the analytes of interest represents a useful strategy for compensation of the matrix effect using matrix-matched calibration standards [50]. A bulk lot of milk matrix was then made from the previously identified blank samples and submitted to spiking experiments to fulfill the matrix-matched approach of the present study and to proceed with the evaluation of the regulated performance criteria.

### 2.1. Method Validation

#### 2.1.1. Method Specificity and Selectivity

The first step of method validation was the assessment of method specificity and selectivity, which was evaluated by comparing individual blank samples from different origins with the corresponding spiked samples. No interfering peaks were observed in the blank matrices (S/N > 3) within a window of ±0.5 min of the retention time (RT) of each analyte, ultimately ensuring proper identification and quantification of the 23 mycotoxins. In the fortified representative blank samples, the analytes of interest were successfully identified without any interference. 

#### 2.1.2. Limits of Detection (LOD) and Limits of Quantification (LOQ)

The background intensity of twenty blank milk sub-samples from the bulk lot was further analyzed at the specific RT for each mycotoxin in order to evaluate the method’s limits by calculating the respective LOD and LOQ. The obtained LOD values ranged between 0.001 and 9.88 ng mL^−1^ for aflatoxin G1 (AFG1) and CIT respectively. For the limits of quantification (LOQ), the values observed were between 0.005 (AFG1) and 13.54 ng mL^−1^ (TEA). The LODs and LOQs are presented in Table 1.

According to the EU regulation [10], as previously mentioned, AFM1 has a maximum allowed level of 0.05 µg kg^−1^ in raw milk, being the only regulated mycotoxin for this foodstuff. The LOD and LOQ values obtained with this method for AFM1 were 0.02 and 0.010 ng mL^−1^, which displays the method’s capacity to detect this mycotoxin at lower concentrations than the maximum level set by the European Commission (EC). For the other AFs, the LOD and LOQ values were also very low, with AFG2 presenting higher values (0.06 ng mL^−1^ for LOD and 0.16 ng mL^−1^ for LOQ). BEA, HT-2, MON, penicillic acid (PA), patulin (PAT), and T-2 toxin had LODs lower than 1 ng mL^−1^, with the higher LODs belonging to CIT and ENNA (9.88 and 9.11 ng mL^−1^, respectively). Other studies on multi-mycotoxin methods, including AFM1, reported values similar to or higher than those achieved in this validation. For example, in a recent study by De Baere et al. [27], the authors validated an UHPLC-MS/MS method for several biological matrices, including cattle milk, to determine aflatoxins B1, B2, G1, G2, and M1. Optimization of different preparation procedures was performed, which encompassed liquid–liquid extraction (LLE), QuEChERS protocols, and SPE, individually or in combination, with a final validation with Oasis^®^ PRiME HLB clean-up SPE. The LOD and LOQ values ranged from 0.002 to 0.038 and 0.025 to 0.5 ng mL^−1^, respectively. A multi-method concerning a wider numerical range of mycotoxins (*n* = 14) in milk has also been recently established by Mao et al. [51], though restricted to AFs, OTs (A and B), ZEA, and its metabolites/derivatives. Using a UHPLC/Q-Orbitrap, the LOD and LOQ values were satisfactorily low, presenting values between 0.0003 and 0.008 ng g^−1^. The first and, to the authors’ knowledge, the only published method for emerging mycotoxins was also recently published for the quantification of a total of 40 mycotoxins using a QuEChERS approach with successful results for the LOD (0.001–3.26 ng mL^−1^) and LOQ (0.002–10.76 ng mL^−1^) parameters [39]. In this study, the LOD and LOQ values for AFM1 were 0.004 and 0.013 ng mL^−1^, respectively. Akinyemi et al. [1] also obtained similar values for AFM1, namely 0.005 and 0.010 ng mL^−1^, respectively, for LOD and LOQ. Concerning other mycotoxins, no maximum levels are established by the European Commission, but they have been reported in raw milk samples, as previously stated [2,12,19,52].

#### 2.1.3. Calibration Curve and Linearity

The matrix-matched approach was then applied by spiking the blank samples at appropriate levels of a multi-standard mycotoxin solution, thus allowing the construction of calibration curves at defined concentration ranges for each mycotoxin. The linearity of the method was evaluated through a linear regression model using the correlation coefficient (R^2^) of the matrix-matched calibration curve in blank raw bulk milk spiked at five concentration levels. The linearity data are presented in Table 1.

The calibration curves are in the ranges as according to Table 1, with maximum levels of 0.2 ng mL^−1^ (AFB1, AFB2, AFG1, AFG2, and AFM1), 20 ng mL^−1^ (DON, HT-2 toxin, NIV, PA, T-2 toxin,), and 200 ng mL^−1^ (BEA, CIT, ENNA, ENNB, mycophenolic acid (MPA), MON, OTA, patulin (PAT), TEA, tentoxin (TTX), and ZEA). The coefficient of correlation values showed good linearities within the broad concentration ranges evaluated, with a maximum value of 0.9996 (HT-2) and a minimum value of 0.9519 (TEA).

Good linearities were therefore achieved, greater than 0.95, with the matrix-matched approach, which was used to compensate for possible effects of matrix interferents in LC-MS/MS [53,54]. These values demonstrate the method’s ability to accurately determine low concentration levels of the mycotoxins in the study.

#### 2.1.4. Precision

Intra-day (repeatability) and inter-day (reproducibility) values were evaluated as coefficients of variation (CV) obtained through the analytical determination of mycotoxins in spiked samples in triplicate on three consecutive days (*n* = 3) at low, medium, and high levels (LL, ML, and HL, respectively). The data are shown in Table 2.

Variations in samples analyzed within the same day (*n* = 3) and on three consecutive days were compliant with the regulatory frameworks, with values lower than 15 and 25% for individual mycotoxins. The acceptance criteria were therefore fulfilled for all compounds at the specific concentration levels according to Commission Regulation (EC) N° 401/2006 [55] and Commission Regulation (EU) N° 519/2014 [56] for regulated mycotoxins and Commission implementing regulation (EU) 2021/808 [57] for non-regulated and emerging mycotoxins. The coefficient of variation values for repeatability ranged from 0.3 to 17.6%, and for reproducibility from 0.6 to 25%. Higher values of precision were obtained for RSD_R_ for the compounds at the lowest concentration level, except for PAT, which presented values of 19.3 (15 ng mL^−1^), 20.8 (100 ng mL^−1^), and 21.1% (200 ng mL^−1^). Nonetheless, the regulated values for PAT are accepted as 30% for 15 and 100 ng mL^−1^ and 25% for 200 ng mL^−1^. The intra-day precision (RSD_r_) values were also below the regulatory values, with FB2, HT-2 toxin, MPA, OTA, and TTX presenting very good RSD_r_ at all concentration levels, in a range of 1.0 to 5.0%.

#### 2.1.5. Recovery

In Table 3, the recovery percentages show a successful extraction of all mycotoxins, in compliance with the regulated performance criteria. The values of recovery expressed as percentages and determined at low, medium, and high concentration levels (LL, ML, and HL, respectively) were within the acceptable ranges with a maximum of 119.9% (ZEA at 20 ng mL^−1^) and a minimum of 67.5% (AFG2 at 0.025 ng mL^−1^).

The recovery values also meet the established European guidelines, between 67.5 and 119.8%. According to Commission Regulation N° 401/2006 and Commission Regulation N° 519/2014 [55,56], the recommended recovery rates for AFB1 and the sum of AFs (B1, B2, G1, and G2) range between 50 and 120%. The values obtained at concentration levels of 0.025, 0.1, and 0.2 ng mL^−1^ were 98.3–100.2% and 91.6–101.7%, respectively. AFM1, FBs, and ZEA also presented values compliant with the regulated range of 60 to 120%; CIT (all levels), DON, T-2, and HT-2 toxins (LL), between the regulated 70–120%; OTA, within 70–110%; and PAT, within 70–105% (LL) and 75–105% (ML and HL). Non-regulated and emerging mycotoxins were evaluated according to CIR 808/2021 [57], which displays the acceptable recovery values for validation purposes at ranges of −50% to +20% for concentrations ≤ 1 ng g^−1^, −30% to +20% for concentrations from >1 ng g^−1^ to 10 ng g^−1^, and −20% to +20% for concentrations ≥ 10 ng g^–1^.

### 2.2. Occurrence of Regulated, Non-Regulated, and Emerging Mycotoxins in Raw Milk

**Figure 1:**
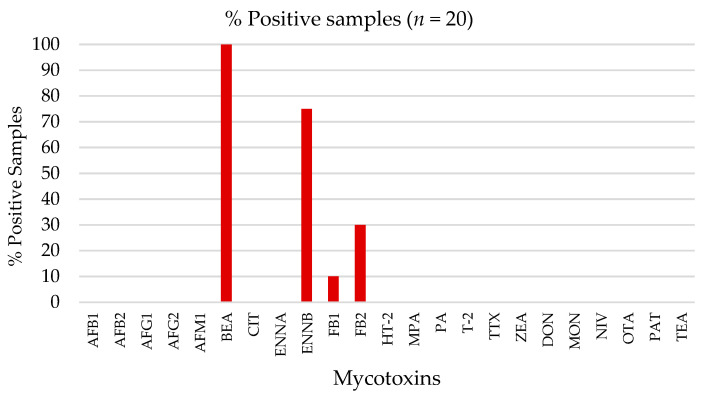
Percentage of positive samples for regulated and emerging mycotoxins in raw milk samples.

To prove the applicability of the method, a total of 20 raw milk samples collected from bulk cooling tanks in the main dairy region of Portugal were subjected to the previous extraction procedure and analyzed for the occurrence of mycotoxins with the validated UHPLC-MS/MS. The occurrence patterns are described in Figure 1.

The emerging mycotoxin BEA was revealed to be the most-commonly occurring mycotoxin in raw milk, with the highest percentages of positive samples, namely 100%. ENNB was also present in 75% of the samples, followed by fumonisins B1 and B2. No AFs were detected, including AFM1. Multi-mycotoxin occurrence data in milk samples are also very scarce. Only very recently have non-regulated and emerging mycotoxins been screened in these food matrices. González-Jartín et al. [39] analyzed 40 mycotoxins in 31 raw milk samples, identifying T-2 toxin, roquefortine C, ENNs, and BEA. These authors also found a high prevalence of the emerging mycotoxins ENNs and BEA, with percentages of approximately 68 and 90%, respectively. In another study, BEA and ENNB were also found in 87.4% and 48.2%, respectively, of a total of 135 milk samples from three different species [1]. Specifically for cow milk, BEA and ENNB occurred in all 23 samples (100%). This work is therefore a new insight on a full screening of several regulated, non-regulated, and emerging mycotoxins in raw milk samples. The high rate of frequency of emerging mycotoxins is aligned with the need for developing new and broader analytical methods to perform occurrence studies based on milk samples, thus allowing the continuous surveillance of all the representative mycotoxins in a single matrix. Ultimately, a precise risk assessment can be designed and applied towards the protection of human health, particularly in vulnerable groups whose milk consumption is high.

## 3. Conclusions

The novelty of the present study was centered on the extension and further validation of a previously optimized analytical method using UHPLC-QTrap-MS/MS for the identification and quantification of 23 regulated, non-regulated, and emerging mycotoxins in raw milk. The validation parameters for the regulated mycotoxins were assessed and validated in consonance with the specifications for confirmatory methods stated in Commission Regulation nº 401/2006; and for non-regulated and emerging mycotoxins, in compliance with ICH guidelines and CIR 808/2021. Good performance criteria were obtained and compliant with the respective regulations, thus displaying its suitability to determine these toxic compounds at low levels. The qualitative characterization of the mycotoxic profiles in raw milk samples as a proof-of-concept also revealed the importance of developing new multi-analyte and multi-matrix methods towards a more comprehensive overview of whole food chains. In this sense, future work should be focused on the integration of new emerging mycotoxins into this laboratory approach, since changing mycotoxic patterns have been occurring worldwide. Limitations regarding the non-regulated mycotoxins and the scarcity of quantification studies of these mycotoxins to prove compliance concerning the limits of detection and limits of quantification of the method, are also important gaps that need to be addressed in order to establish proper risk assessments on this recognized public health risk.

## 4. Materials and Methods

### 4.1. Chemicals and Reagents

Reagents of analytical grade were mainly used, with the exception of mobile-phase reagents, which comprised solvents of high-performance liquid chromatography (HPLC) grade. Analytical standards of MPA and NIV were purchased from Supelco (Bellefonte, PA, USA); of PA, from Santa Cruz Biotechnology (Dallas, TX, USA); and of AFB1, AFB2, AFG1, AFG2, AFM1, BEA, CIT, DON, ENNA, ENNB, FB1, FB2, HT-2, T-2 toxins, MON, OTA, PAT, TEA, TTX, and ZEA from Sigma-Aldrich (Steinheim, Germany). Ultrapure H_2_O was supplied through a Milli-Q water system from Merck (Saint-Quentin-Fallavier, France) and C18 sorbent from Agilent Technologies (Santa Clara, CA, USA). Acetonitrile (ACN) was acquired from Carlo Erba (Val de Reuil, France), anhydrous magnesium sulfate (MgSO_4_) and sodium chloride (NaCl) from Honeywell (Seelze, Germany), and formic acid from Chem-Lab (Zedelgem, Belgium). ACQUITY UPLC^®^ HSS T3 1.8 μm (2.1 × 100 mm i.d.) was obtained from Waters (Milford, MA, USA); HPLC vials and Syringeless Device Mini UniPrep filters (0.45 µm PVDF, polypropylene), from Whatman (Maidstone, England).

### 4.2. LC-MS/MS Parameters

Mycotoxin determination in milk samples was performed using a UHPLC Nexera X2 Shimadzu system (Shimadzu, Kyoto, Japan) coupled to a Triple QTRAP 5500+ detector (Sciex, Foster City, CA, USA). For compound separation, an ACQUITY UPLC^®^ HSS T3 1.8 μm (2.1 × 100 mm i.d.) column was used and the sequential mass detector (UHPLC-MS/MS) operated in a single run in positive and negative ion mode in a single run (ESI+/ESI−) through an electrospray interface (Turbo Ion Spray). Multiple Reaction Monitoring (MRM) parameters were previously defined for each compound, as well as ion transitions [38]. LC-MS/MS parameters were as follows: injection volume, 20 μL; temperatures of the column and autosampler, 30 °C and 10 °C, respectively; flow rate, 0.2 mL min^−1^; mobile-phase composition and gradient elution program: (A) 0.1% formic acid and (B) acetonitrile; gradient elution protocol, 95% A to 30% A (15 min), 30% A to 0% A (5 min, 2 min hold), 0% A to 95% A (3 min); run time: 25 min. Software for data acquisition and processing comprised Analyst^®^ and MultiQuant^TM^ (Sciex, Foster City, CA, USA).

### 4.3. Preparation of Calibration Standards and Fortified Samples

Individual standard solutions were prepared in ACN 100% (*v*/*v*) for the following mycotoxins: AFB2, AFG1, AFM1, BEA, CIT, DON, ENNA, ENNB, HT-2, T-2 toxins, MON, MPA, NIV, PA, PAT, TEA, TTX, and ZEA. For AFB1, AFG2, and OTA, standard solutions were prepared in MeOH 100% (*v*/*v*), and for FBs the standard preparation was performed in ACN:H_2_O (50:50, *v*/*v*). Stock solutions of 1 mg mL^−1^ were prepared for all mycotoxins with the exception of T-2 toxin, which was prepared at a concentration of 2.5 mg mL^−1^. A multi-standard final solution, for quality control (QC) fortification procedures, was prepared in ACN:H_2_O (80:20, *v*/*v*) using working solutions that were prepared by diluting individual standard solutions in ACN 100% (*v*/*v*). All solutions were kept in the dark at −20 ± 2 °C in amber vials. For the validation procedure, fortification of blank milk samples (4.0 ± 0.1 mL) was performed by adding appropriate quantities of the multi-standard solution to the initial samples.

### 4.4. Milk Samples

Raw milk samples were obtained from dairy farms of the main dairy region of Portugal in the years 2020 and 2021. Sampling was performed directly from bulk milk cooling tanks into sterile, labeled, screw-top bottles into a final volume of 1 L in order to comply with Commission Regulation (EC) nº 401/2006 [55]. The samples were stored individually at −20 ± 2 °C until further analysis. Prior to method validation, the samples were analyzed to search for blanks to be used as quality control (QC) by spiking them with multi-mycotoxin standards. The identified blank samples were further combined to obtain a single representative blank bulk milk sample by manual shaking. Aliquots of the bulk were further analyzed to guarantee a correct homogenization process through the analysis of precise S/N ratios in each aliquot. The representative bulk milk was finally submitted to the extraction procedure for performance criteria validation.

### 4.5. Extraction of Mycotoxins from Milk

Extraction of mycotoxins from raw milk was performed as previously described by Leite et al. [38]. A 16 mL volume of ACN:H_2_O (80:20, *v*/*v*) was added to the raw milk matrices (4.0 ± 0.1 mL), and homogenization for 60 min at room temperature was performed on a horizontal shaker. The partitioning step of the modified QuEChERS protocol consisted of 0.5 g of NaCl and 2.0 g of MgSO_4_ (1:4, *w*/*w*), which was combined with the previous solution and stirred for 1 min, then further centrifuged for 10 min at 4 °C and 4500× *g*. A 10 mL volume of the upper layer was collected and 150 mg C18 and 900 mg MgSO_4_ were added for dSPE purposes. A second centrifugation was performed under the same conditions as previously, and the extract was submitted to a complete drying process under a nitrogen stream following the use of a Turbovap Zymark Evaporator system from Biotage (Hopkinton, MA, USA). A reconstitution step of the final extract was conducted in 40% ACN at a volume of 500 µL, with further filtration in HPLC vials and injection in the UHPLC-MS/MS system.

### 4.6. Method Validation

Validation of the present method in milk samples, which aimed at the qualitative and quantitative determination of 23 regulated, non-regulated, and emerging mycotoxins in such matrices, was performed in compliance with the performance criteria guidelines defined by the European Commission (EC), European Medicines Agency (EMA), and Food and Drug Administration (FDA) [55,56,57,58,59]. The selectivity and specificity were assessed by analyzing blank samples at the corresponding R.T. and, simultaneously, corresponding blank samples spiked with a multi-standard solution to evaluate possible peak interferences. Twenty QC sub-samples were also evaluated as signal-to-noise 3:1 and 10:1 for LOD and LOQ determination, respectively. The calibration curves were constructed with five calibration points to assess the method’s linearity through a matrix-matched approach and evaluated with the method of least squares for each analyte and the respective correlation coefficients (R^2^). The precision of the method was analyzed in triplicate on three consecutive days at three concentration levels, which comprised spiking of QC at a low concentration level (LL), medium concentration level (ML), and high concentration level (HL). Intra- and inter-day variation were expressed as coefficients of variation calculated on the basis of Equation (1):

CV (%) = σ/µ × 100(1)

where σ is the standard deviation at each calibration level and µ is the mean concentration.

The analysis of QC at the three aforementioned levels (LL, ML, and HL) also allowed the assessment of the performance criteria trueness through the determination of extraction recoveries obtained by calculating Equation (2):

Recovery (%) = A_ex_/A_th_ × 100 (2)

where A_ex_ is the average concentration of replicates and A_th_ is the theoretical concentration assayed.

## Figures and Tables

**Table 1:** Performance criteria for mycotoxins in milk: linearity, limits of detection (LOD), and limits of quantification (LOQ).

Mycotoxin	Concentration Range (ng mL^−1^)	Linearity (R^2^)	LOD (ng mL^−1^)	LOQ (ng mL^−1^)
AFB1	0.025–0.2	0.9982	0.005	0.015
AFB2	0.025–0.2	0.9969	0.002	0.006
AFG1	0.025–0.2	0.9979	0.001	0.005
AFG2	0.025–0.2	0.9678	0.060	0.16
AFM1	0.025–0.2	0.9976	0.002	0.010
BEA	5–200	0.9882	0.74	2.28
CIT	15–200	0.9651	9.88	10.77
DON	2.5–20	0.9786	1.25	3.84
ENNA	10–200	0.9830	9.11	9.32
ENNB	10–200	0.9900	4.22	4.35
FB1	10–200	0.9925	0.68	3.08
FB2	10–200	0.9948	0.40	5.50
HT-2	1–20	0.9996	0.003	0.08
MON	5–200	0.9911	0.66	1.28
MPA	15–200	0.9961	4.10	11.88
NIV	15–200	0.9862	4.10	12.12
OTA	10–200	0.9910	4.26	7.17
PA	1–20	0.9893	0.04	0.13
PAT	15–200	0.9908	0.59	8.44
T-2 toxin	2–20	0.9989	0.31	0.87
TEA	15–200	0.9519	3.83	13.54
TTX	15–200	0.9963	4.00	11.60
ZEA	15–200	0.9607	2.34	8.65

AFB1—Aflatoxin B1; AFB2—Aflatoxin B2; AFG1—Aflatoxin G1; AFG2—Aflatoxin G2; AFM1—Aflatoxin M1; BEA—Beauvericin; CIT—Citrinin; DON—Deoxynivalenol; ENNA—Enniatin A; ENNB—Enniatin B; FB1—Fumonisin B1; FB2—Fumonisin B2; MON—Moniliformin; MPA—Mycophenolic acid; NIV—Nivalenol; LOD—Limit of Detection; LOQ—Limit of Quantification; OTA—Ochratoxin; PA—Penicillic Acid; PAT—Patulin; TEA—Tenuazonic acid; TTX—Tentoxin; ZEA—Zearalenone.

**Table 2:** Performance criteria for mycotoxins in milk: repeatability (RSD_r_) and reproducibility (RSD_R_) expressed as coefficient of variation (CV) in percentage.

Mycotoxin	Concentration (ng mL^−1^)	RSD_r_ (%)	Regulated RSD_r_ (%) ^1^	RSD_R_ (%)	Regulated RSD_R_ (%) ^1^	
AFB1	0.025	6.6	20	9.7	30	
	0.1	4.6		4.6		
	0.2	1.8		5.6		
AFB2	0.025	7.8	-	10.4	-	
	0.1	3.0		4.7		
	0.2	3.4		4.2		
AFG1	0.025	0.3	-	4.5	-	
	0.1	1.4		1.4		
	0.2	2.6		5.3		
AFG2	0.025	13.8	-	19.7	-	
	0.1	5.0		19.9		
	0.2	4.3		10.4		
	0.025	17.6	20	15.2	30	
Sum of AFs	0.1	2.5		1.9		
	0.2	1.0		0.6		
AFM1	0.025	8.3	20	7.2	30	
	0.1	1.7		2.6		
	0.2	1.7		2.5		
BEA	5	9.1	17	10.4	25	
	100	5.8		12.5		
	200	10.5	15	13.2	22	
CIT	15	13.6	17	10.6	50	
	100	9.1		13.9		
	200	4.9	15	7.0	44	
DON	2.5	12.6	20	25.0	30	
	10	12.5	17	10.6	25	
	20	8.0		7.5		
ENNA	10	12.6	17	18.8	25	
	100	5.3		16.3		
	200	9.5	15	7.1	22	
ENNB	10	8.2	17	6.1	25	
	100	2.9		11.1		
	200	7.6	15	9.9	22	
FB1	10	4.8	30	14.87	60	
	100	2.7		7.93		
	200	0.04		3.92		
FB2	10	2.1	30	8.6	60	
	100	1.5		5.2		
	200	1.6		3.8		
HT-2	1	4.9	20	5.9	30	
	10	1.3	17	4.8	25	
	20	1.3	30	3.7	50	
MON	5	12.7	17	11.6	25	
	100	8.1		19.3		
	200	3.6	15	18.1	22	
MPA	15	5.0	20	4.9	30	
	100	0.6	17	2.3	25	
	200	0.4		1.8		
NIV	15	12.4	20	19.6	30	
	100	1.9	17	12.8	25	
	200	2.5		5.9		
OTA	10	3.8	20	8.6	30	
	100	4.3		5.0		
	200	2.2		2.3		
PA	1	8.8	20	7.9	30	
	10	1.9		2.5		
	20	1.0		1.6		
PAT	15	14.2	20	19.3	30	
	100	6.7	15	20.8	25	
	200	7.1		21.1		
T-2 toxin	2	11.6	20	9.7	30	
	10	2.0	17	8.2	25	
	20	1.2	30	4.5	50	
TEA	15	8.0	20	22.6	30	
	100	15.3	17	18.7	25	
	200	4.1		16.4		
TTX	15	1.2	20	2.4	30	
	100	1.8	17	2.8	25	
	200	1.0		2.1		
ZEA	15	7.8	40	12.8	50	
	100	6.3		8.2		
	200	11.5		6.8		

^1^ Adapted from Commission Regulation (EC) N° 401/2006 and Commission Regulation (EU) N° 519/2014 (regulated mycotoxins); and from Commission implementing regulation (EU) 2021/808 (non-regulated and emerging mycotoxins). AFB1—Aflatoxin B1; AFB2—Aflatoxin B2; AFG1—Aflatoxin G1; AFG2—Aflatoxin G2; AFM1—Aflatoxin M1; AFs—Aflatoxins (B1, B2, G1, and G2); BEA—Beauvericin; CIT—Citrinin; DON—Deoxynivalenol; ENNA—Enniatin A; ENNB—Enniatin B; FB1—Fumonisin B1; FB2—Fumonisin B2; MON—Moniliformin; MPA—Mycophenolic acid; NIV—Nivalenol; OTA—Ochratoxin; PA—Penicillic Acid; PAT—Patulin; TEA—Tenuazonic acid; TTX—Tentoxin; ZEA—Zearalenone.

**Table 3:** Performance criteria for mycotoxins in milk: Recovery (%).

Mycotoxin	Concentration Range (ng mL^−1^)	Recovery (%)			Regulated Recovery (%)
		LL	ML	HL	
AFB1	0.025–0.2	100.2	98.3	99.7	50–120
AFB2	0.025–0.2	106.1	101.7	98.5	-
AFG1	0.025–0.2	106.3	101.9	97.8	-
AFG2	0.025–0.2	67.5	104.1	100.2	-
Sum of AFs	0.025–0.2	91.6	101.7	99.1	50–120
AFM1	0.025–0.2	107.8	102.7	98.1	60–120
BEA	5–200	94.8	104.7	98.3	80–120
CIT	15–200	94.9	76.6	107.3	70–120
DON	2.5–20	71.3	111.4	94.8	70–120 (>1–10) 80–120 (≥10)
ENNA	10–200	117.9	94.8	99.4	80–120
ENNB	10–200	112.7	94.7	100.4	80–120
FB1	10–200	114.0	109.7	96.7	60–120
FB2	10–200	114.6	93.0	101.7	60–120
HT-2	1–20	99.6	101.7	99.8	60–130
MON	5–200	106.5	116.8	93.9	80–120
MPA	15–200	101.0	105.9	97	70–120 (>1–10) 80–120 (≥10)
NIV	15–200	76.3	103.7	100.1	70–120 (>1–10) 80–120 (≥10)
OTA	10–200	108.5	88.9	104.5	70–110
PA	1–20	75.2	110.1	95.8	50–120
PAT	15–200	90.8	105.5	98.8	50–120 (<20) 70–105 (20–50) 75–105 (>50)
T-2 toxin	2–20	96.3	100.4	99.1	60–120
TEA	15–200	79.8	112.0	99.6	70–120 (>1–10) 80–120 (≥10)
TTX	15–200	101.4	105.4	96.4	70–120 (>1–10) 80–120 (≥10)
ZEA	15–200	119.8	94.4	99.7	60–120

AFB1—Aflatoxin B1; AFB2—Aflatoxin B2; AFG1—Aflatoxin G1; AFG2—Aflatoxin G2; AFs—Aflatoxins (B1, B2, G1, and G2); AFM1—Aflatoxin M1; BEA—Beauvericin; CIT—Citrinin; DON—Deoxynivalenol; ENNA—Enniatin A; ENNB—Enniatin B; FB1—Fumonisin B1; FB2—Fumonisin B2; MON—Moniliformin; MPA—Mycophenolic acid; NIV—Nivalenol; LL—Low Level; ML—Medium Level; HL—High Level; OTA—Ochratoxin; PA—Penicillic Acid; PAT—Patulin; TEA—Tenuazonic acid; TTX—Tentoxin; ZEA—Zearalenone.

## Data Availability

Data sharing is not applicable to this article.
